# Correction: Mudie et al. In Vitro-In Silico Tools for Streamlined Development of Acalabrutinib Amorphous Solid Dispersion Tablets. *Pharmaceutics* 2021, *13*, 1257

**DOI:** 10.3390/pharmaceutics13122059

**Published:** 2021-12-02

**Authors:** Deanna M. Mudie, Aaron M. Stewart, Jesus A. Rosales, Molly S. Adam, Michael M. Morgen, David T. Vodak

**Affiliations:** Global Research & Development, Lonza, Bend, OR 97703, USA; aaron.stewart@lonza.com (A.M.S.); rosaleja@uw.edu (J.A.R.); molly.adam@lonza.com (M.S.A.); michael.morgen@lonza.com (M.M.M.); david.vodak@lonza.com (D.T.V.)

The authors wish to make the following corrections to this paper [1].

There was an error in the original article in the Abstract section, where it was stated that the absolute average fold error (AAFE) of the in silico predictions for AUC_0-inf_ for Calquence + famotidine was ≈3. A correction has been made to the abstract section: “In silico simulations of the HPMCAS-H ASD tablet and Calquence capsule provided good in vivo study prediction accuracy using a bottom–up approach (absolute average fold error of AUC_0-inf_ < 2)”.

There was an error in the original article in the Results section (Section 3.6, paragraph 1), where it was stated that AAFE of the in silico predictions for AUC_0-inf_ for the Calquence capsule + famotidine treatment was not <2. A correction has been made in the original article in the Results section (Section 3.6, paragraph 1): “The AAFE of the in silico predictions for AUC_0-inf_, C_max_, T_max_, and C_p_ versus time for all formulation treatments were <2-fold (ideal value = 1) with the exception of C_p_ versus time for the Calquence capsule + famotidine treatment, indicating that the in silico prediction framework is sufficient for simulating acalabrutinib blood plasma concentrations within this in vivo study”.

In the original article, there were mistakes in Table 4 as published, where AAFE for AUC_0-inf_ for the Calquence capsule + pentagastrin was listed as 1.2 and AAFE for AUC_0-inf_ for Calquence capsule + famotidine was listed as 3.6. The corrected Table 4 appears below.

The authors apologize for any inconvenience caused and state that the scientific conclusions are unaffected. The original publication has also been updated.

## Figures and Tables

**Table 4:** Noncompartmental analysis comparing simulated (sim) versus observed (obs) data for all formulation treatments in the dog study. Absolute average fold error (AAFE) was calculated for AUC_0-inf_, C_max_, T_max_, and C_p_ versus time to determine the accuracy of the in silico prediction exercise (ideal value = 1).

Formulation	AUC_0-inf_ (ng h/mL)		C_max_ (ng/mL)		T_max_ (h)		AAFE			
	Obs	Sim	Obs	Sim	Obs	Sim	AUC_0-inf_	C_max_	T_max_	C_p_ vs. Time
ASD tablet, pentagastrin	8161	9766	3332	3727	0.9	0.9	1.2	1.2	1.6	1.3
ASD tablet, famotidine	7579	9555	3443	3508	0.9	1.6	1.3	1.2	1.8	1.6
Calquence capsule, pentagastrin	8365	8607	4480	3110	0.8	0.9	1.1	1.4	1.3	1.3
Calquence capsule, famotidine	3112	3096	355	648	1.6	1.2	1.6	1.9	1.7	3.0
